# Effectiveness of implementing evidence-based approaches to promote physical activity in a Midwestern micropolitan area using a quasi-experimental hybrid type I study design

**DOI:** 10.1186/s12889-024-18523-9

**Published:** 2024-04-19

**Authors:** Barbara Baquero, Nicole Novak, Daniel K. Sewell, Christine M. Kava, Jason Daniel-Ulloa, Hanh Pham, Natoshia Askleson, Sato Ashida, Helena Laroche, Adriana Maldonado Gonzalez, Rebecca Bucklin, Heidi Haines, Edith A. Parker, The Active Ottumwa Community Advisory Board, Lynelle Diers, Himar Hernandez, Kim Hellige, Brian Dunn, Gene Rathje, Claudia Gates, Garrett Ross, Rebecca Ellingson

**Affiliations:** 1 Department of Health Systems and Population Health, University of Washington School of Public Health, Seattle, WA USA; 2 Department of Community and Behavioral Health, University of Iowa College of Public Health, Iowa City, IA USA; 3 University of Iowa Prevention Research Center for Rural Health, Iowa City, IA USA; 4 Department of Biostatistics, University of Iowa College of Public Health, Iowa City, IA USA; 5 Department of Health Systems and Population Health, University of Washington, School of Public Health, Seatle, WA USA; 6 Center for Children’s Healthy Lifestyles and Nutrition, Children’s Mercy Kansas City, Kansas City, MO USA

**Keywords:** Physical activity, Evidence-based, Community-based participatory research, Participatory implementation

## Abstract

**Background:**

Much evidence-based physical activity (PA) interventions have been tested and implemented in urban contexts. However, studies that adapt, implement, and evaluate the effectiveness of these interventions in micropolitan rural contexts are needed. The study aimed to evaluate the effectiveness of the Active Ottumwa intervention to promote PA in a micropolitan community.

**Methods:**

Between 2013 – 2019, we implemented Active Ottumwa in a micropolitan setting, and subsequently implemented and evaluated its effectiveness using a Hybrid Type I design. In this paper, we describe the intervention’s effectiveness in promoting PA. We collected PA data over 24 months from a cohort of community residents using accelerometers and PA data from two cross-sectional community surveys administered in 2013 and 2018, using the Global Physical Activity Questionnaire.

**Results:**

From the cohort, we found significant change in PA over 24 months (*P* = 0.03) corresponding to a 45-min daily decrease in sedentary activity, a daily increase of 35-min in light PA and 9 min in moderate-to-vigorous PA. There was a statistically significant (*P* = 0.01) increasing trend at the population-level in the moderate-to-vigorous composition of 7 min between the two cross-sectional assessments (95% CI: 0.1%—1.34%).

**Conclusions:**

The study demonstrates that the adapted evidence-based PA interventions in a micropolitan context is effective.

**Supplementary Information:**

The online version contains supplementary material available at 10.1186/s12889-024-18523-9.

## Background

Despite advances in identifying effective interventions to promote physical activity (PA), the percentage of people meeting PA recommendations remains low. In 2018, only 22.9% of adults 18–64 living in the US met both aerobic and muscle-strengthening PA guidelines [1–3]. The health benefits of greater levels of PA are well documented [4–6]. Evidence supports that any amount of activity is beneficial [7]. Low intensity or light PA have been associated with pain reduction, increased flexibility, muscle strengthening, lower risk of all-cause mortality and cardiovascular mortality, lower and regulated insulin and glucose, and improved mental health [4, 8, 9]. Small increases in minutes and intensity of PA can also have positive health effects [8]. For example, replacing 30 min a day of sedentary behavior with light PA reduces the risk for cardiovascular disease [9, 10] and type 2 diabetes [11, 12]. Continuing to examine how to promote and expand on PA opportunities can have a meaningful impact on the health of the population. This may be particularly important in rural areas, where patterns of inactivity are greater [13].

PA rates among people living in rural areas are even lower. Interventions to promote PA in rural areas are limited and less conclusive [14]. Residents living in rural communities experience worse health outcomes associated with physical inactivity. compared to residents in urban areas [2, 3, 8]. The lack of opportunities for PA in rural areas is associated in part with limited resources (e.g., smaller budgets for health promotion services), lack of trained community leaders, geography (e.g., greater distances between destinations), values and beliefs (e.g., strong social networks, and individualistic mindsets) that characterize rural residents and rural areas.

Many interventions promoting PA at the population level have been tested and implemented in urban contexts [14–16]. However, studies that adapt, implement, and evaluate the effectiveness of these interventions in micropolitan (i.e., non-metropolitan areas centered socially and economically around a population core of 10,000–50,000 people) contexts are needed [17]. We developed Active Ottumwa, a community-level intervention to promote PA in a micropolitan community in the rural United States.

This paper describes the findings from the evaluation of Active Ottumwa. We used a longitudinal cohort survey to assess individual changes in PA from baseline to 24 months, and cross-sectional community surveys to assess population-level PA changes before and after the intervention. We hypothesized that adapting and implementing evidence-based approaches to increase PA in a micropolitan community would significantly increase minutes of PA.

## Methods

### Study overview and aim

**Fig. 1:**
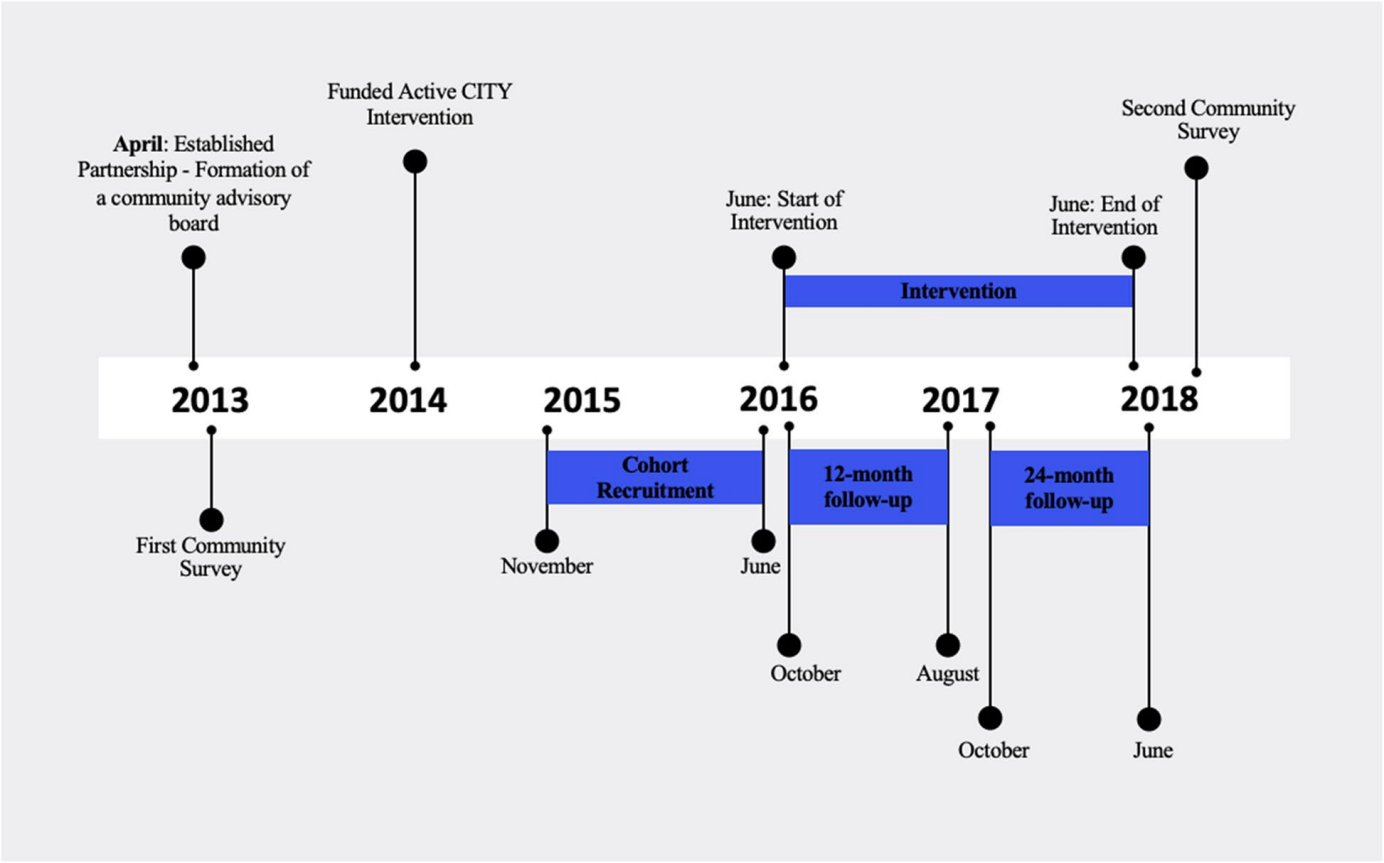
Study timeline: sequence of activities and benchmarks of the study

The full study applied a Hybrid Type 1 design, which is a study design that test the effect of the intervention and its delivery in the real-world [18], in this paper we focus on reporting the effectiveness of the intervention. The primary outcome of the study was the number of minutes of PA per week, measured by accelerometer data from the cohort sample at baseline, 12 months and 24 months, and by self-report in the cross-sectional community sample. Figure 1 depicts the study timeline and major activities.

### Setting

Ottumwa, the micropolitan community where this study intervened has a population of nearly 25,000 [19]. The county where the micropolitan area is located is ranked 97 out of 99 counties in both overall health outcomes and health factors, with high rates of premature death (8,700 vs 6,500 for Iowa and 7,300 for the US of years of potential life lost before age 75 per 100,000 people), obesity (38% vs 34% vs 32% respectively), and physical inactivity (30% vs 26% and 26% respectively) [20]. By the time of the intervention, there had been a 1600% growth in Latino residents in Ottumwa between 1990 (200 Latino residents) and 2016 (3401 Latino residents), and Latinos now make up 14% of the town’s population. Ottumwa also has higher rates of poverty (16.6%) compared to both the state (11.2%) and the United States (10.5%) [20].

### Active Ottumwa intervention design

A Community-Based Participatory Research (CBPR) approach guided the adaptation, implementation and evaluation of the intervention [21]. Intervention approaches for PA were selected from the Community Guide for Preventive Services [15]. Table 1 describes each approach level, intervention activities under each approach and an example of the activities conducted. In summary, for the campaign and information approach, a community-wide media campaign was selected and implemented to increase awareness and engagement with the intervention activities, a website, and social media presence was created, and face-to-face promotional activities were conducted. At the behavioral and social approach level, we recruited, trained, and supported volunteer community members as lay health advisors (LHA), to implement group PA activities across the community. For the environmental and policy approach level, we collaborated with local city officials and other community organizations to support initiatives and policies that promote active living and PA such as exercise groups and urban design policies. Further details on our intervention were published elsewhere [22]. The intervention was implemented from June 2016 to June 2018.

**Table 1:** Intervention approaches, adapted activities and examples PA activities in the community

Evidence-based approach	Adapted Activities	Example
Campaign and informational	Increasing awareness of the program across the city	Mass media campaign through local and trusted channels, such as movie theaters, local newspapers, and social media
Behavioral and social	Recruiting and training LHA	Recruited LHA from diverse settings and social groups, trained, and supported them to start and maintain a PA group
Environmental and policy	Collaborating with local official and community-based organizations to advance structural changes that supported PA	Attend local city council meetings to advocate for maintenance of public spaces, work with local organizations to maintain open spaces for PA

*LHA* Lay Health Advisor, *PA* Physical Activity

### Study design and sampling

We used longitudinal cohort (interviews) and a cross-sectional (surveys) research designs to collect data to measure effectiveness. We used random digit dialing of both landline and cell phone numbers to recruit community residents for the longitudinal cohort study and the two cross-sectional community surveys [23]. Eligibility criteria included: being between the ages of 18 and 68, have lived in the community for at least the past six months, and planning to stay or live in Ottumwa for the next two years (the length of the intervention). To understand the impact of the intervention on the growing Latino community, Latino residents were oversampled in the recruitment process. The application of this recruitment method is further described in our previous publication [22].

### Data procedures and analysis

#### Longitudinal cohort study data collection procedures

Trained research assistants collected data using the Research Electronic Data Capture system hosted by the Midwestern university [24]. After completing the in-person interview, participants wore an accelerometer on their non-dominant wrist continuously for seven days, including at night. Participants were instructed to remove the accelerometer eight days following their office visit and return it via pre-paid mail envelope that was provided at the visit. A similar protocol was also collected from cohort participants at 24 months. Participants received $25 after completing their visit measures.

At baseline, 12- and 24-months data collection points, we called participants up to eight times via phone to schedule an appointment. Our data collection points occurred on a rolling basis. Therefore, if a participant completed baseline in 2015 at the beginning of our enrollment period, their 12-month follow-up will occur in 2016 and 24 months in 2017, our Fig. 1 depicts. We sent a postcard if we were unable to make phone contact. To retain our cohort participants, check-in follow-up calls were conducted at six, 15 and 18 months.

#### Longitudinal cohort study measures and variables

Self-reported demographic characteristics (gender, sex, age, education, race/ethnicity) were collected during the baseline interview and included in this analysis. Participants also reported whether they had seen any messages or advertising promoting PA in the past 6 months; whether they had heard of Active Ottumwa; whether they had begun doing more PA as a result of Active Ottumwa messages; whether they had participated in a PA event with a LHA; whether they had heard of or attended specific LHA events (e.g. walking groups, Zumba, tai chi). The primary outcome variable was collected using the ActiGraph GT9X accelerometer (ActiGraph LLC, Pensacola, FL) [25] to collect objective PA minutes data. In Metcalf et al. we published the calibration and data analysis details of our accelerometry measure. In sum, our epoc length was + /_ 8 units and epoc frequency was collected at a rate of 80 Hz. Participants worn the accelerometer for 7 days and we consider a valid day of at least 10 h of wear. Cut points for levels of PA were calculated during the calibration study using a series of decision tree algorithms [26]. The variable was a compositional slope score describing each cohort participant’s PA at baseline and 24 months. We computed the compositional slope score by determining each participant’s PA minutes as sedentary activity (SA), light physical activity (LPA), and moderate-to-vigorous physical activity (MVPA), then these values were scaled by the ttal wear time to obtain each participant proportion of time spent in each of these categories at baseline and 24-months [27]. This data analatic was selected to account for the time each participant was exposed to the intervention.

Exposure to the Active Ottumwa intervention was measured by the amount of time spent participating in the intervention between baseline and 24-month follow-up. Due to a rolling recruitment period, the amount of time between baseline and 24-month follow-up that was spent under exposure to the Active Ottumwa intervention differed between participants, with some participants enrolled several months prior to the start of the intervention implementation, and others recruited after the intervention had started. We calculated the amount of time exposed to the intervention for each participant (i.e., time of 24-month follow-up minus the maximum of either the time from baseline data collection or the start of the intervention).

#### Longitudinal cohort study data analysis

To evaluate the effectiveness of Active Ottumwa, we analyzed the change in PA composition (SA, LPA, and MVPA) between baseline and 24-month follow-up. There were four steps to our primary analysis. These steps are outlined below; for details, see Supplementary file 1.

First, we computed the compositional score slope, a measure of the change in an individual’s SA, LPA, and MVPA over the time exposed to the intervention. There is a conceptual equivalence between our compositional slopes and traditional temporal slopes in that they both measure change over time, but because they were computed in such a way that respected the compositional aspect of our data, numerically these compositional slopes are less easily interpretable. An important special case, however, is when the slope equals (1/3, 1/3, 1/3) which corresponds to no change over time. The precise definition of and more discussion on the compositional slope is given in the Supplementary file 1 [26–28].

Second, we performed centered log ratio (clr) transformations of the compositional slopes. The clr transform is a standard approach to enable the use of more classic statistical procedures on compositional data. Note that the clr of a slope which corresponds to no change over time is (0, 0, 0).

Third, we performed multivariate analysis of variance (MANOVA) on the clr variables. The MANOVA model was adjusted for the following covariates assessed by self-report in the baseline survey: age (in years), gender (male, female), and education (8th grade or less, some high school, graduated high school, or some college or more). We also included the clr transformed baseline PA variables to account for possible regression to the mean (e.g., those with higher-than-average SA at baseline would be expected to show a decrease in SA over time back towards the average). To estimate a global change in PA, we centered our continuous-valued covariates and used sum-to-zero contrasts for the factor variables, leaving the intercept in the model to capture the desired global compositional slope. That is, if the intercept in the MANOVA model is zero for SA, LPA, or MVPA, then any change over time for the respective component of the composition is solely due to the confounding factors such as age or gender. We tested the significance of all of the variables in our model using the Pillai-Bartlett test.

Fourth, given baseline PA measurements and a compositional slope, we computed directly the endline measurements. Therefore, to make the results more interpretable we used archetypal baseline PA values in conjunction with the mean compositional slope estimated from the MANOVA model to compute expected endline PA. The resulting compositions were also translated into number of minutes out of a 16 h awake period. We selected a 16- hour day to account for participants who worked third shifts in the area’s food processing and manufacturing plants.

We restricted the analysis to individuals with baseline and 24-month data and complete data on age, gender and education. We used the compositional variable describing each subject’s PA to compute the compositional mean PA for baseline and 24 months.

#### Cross-sectional community surveys data collection procedures

For both community surveys in 2013 and 2018, participants were called up to 10 times at different times of the day and days of the week to complete interviews. Interviews were conducted in English or Spanish.

#### Cross-sectional community surveys measures and variables

The Global Physical Activity Questionnaire (GPAQ) was used to assess PA levels. The questionnaire measures weekly minutes of MVPA and sedentary behavior. We selected this questionnaire because it had been validated at a population level, could be completed over the phone, and would allow us to compare PA levels between community and national samples [29].

In a previous study, we found the raw GPAQ values to have a weak relationship with true levels of PA [26]. We used methods developed in our calibration study to analyze the GPAQ in the surveys to obtain a more accurate measure of PA. The details on methods were published by Metcalf et al. [26].

#### Cross-sectional community surveys data analysis

To test changes in PA at the population level over the intervention period we used the clr transformation of both the GPAQ and recalibrated GPAQ values. To obtain population mean estimates, we first used American Community Survey (ACS) 5-year estimates for the city’s population in 2013 and 2017 to create sampling weights stratified on age, gender, and ethnicity; age was polychotomized to match available ACS data. We then estimated for both 2013 and 2018 surveys the population mean clr variables using a weighted average and computed their corresponding standard errors [28]. The change in mean clr variables between 2013 and 2018 was evaluated using a Wald test, and we created confidence intervals on the mean change in the composite scale using the Delta method. See Supplemental file 2 for more details.

## Results

### Longitudinal cohort study

**Fig. 2:**
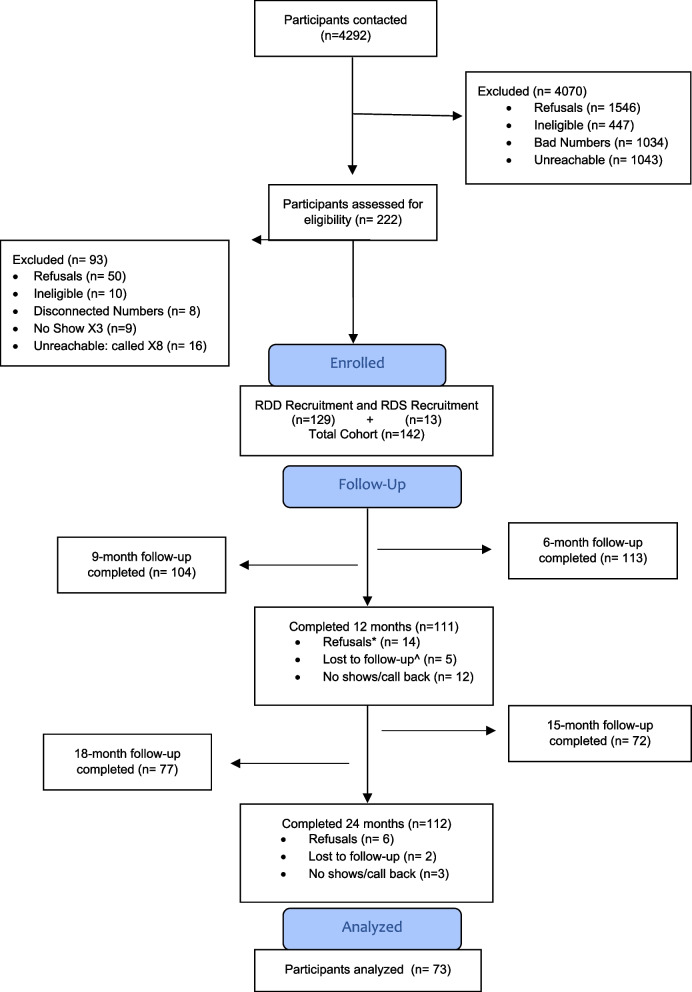
CONSORT flowchart for intervention studies: Active Ottumwa 2013–2019

We contacted 4,292 people of which 222 were eligible and 142 residents consented to participate in the cohort. For details see Fig. 2. We collected baseline data from 142 participants. We excluded participants missing accelerometry data (*n* = 21), demographic data (*n* = 1), or 24-month follow-up data (*n* = 47), for a final analytic sample of 73 participants.

Table 2 shows the demographics of the cohort used in our analysis as well as their baseline and follow-up mean PA levels; 66% were female, 68% had completed at least some college, 82% were non-Latino White and 15% were Latino, and the mean age was 52 years. The average (SD) time between baseline and follow-up visits was 2.0 (0.11) years. At the 24-month follow-up, 99% of participants reported having heard of Active Ottumwa, 93% reported seeing messages or advertising to promote PA in the past 6 months and 19% reported participating in at least one Active Ottumwa event.

**Table 2:** Demographics and physical activity statistics of cohort members (*n* = 73): Ottumwa, Wapello, Iowa, 2013–2019

	**N (%)**	**Mean (sd)**	**Compositional Mean**
Gender			
Female	48 (66%)		
Male	25 (34%)		
Education			
Less than high school	4 (5.5%)		
Some high school	4 (5.5%)		
Graduated high school	15 (21%)		
Some college or more	50 (68%)		
Race/ethnicity			
Non-Latino White	60 (82%)		
Non-Latino Black	1 (1%)		
Other or Multiracial (Non-Latino)	1 (1%)		
Latino	11 (15%)		
Age		52 (13)	
Baseline Sedentary^a^			0.7506
Baseline Light^a^			0.1715
Baseline MVPA^a^			0.0779
24-Month Sedentary^a^			0.7469
24-Month Light^a^			0.1804
24-Month MVPA^a^			0.0727
At 24 months, has seen, read or heard any messages to promote PA in the community in the past 6 months	68 (93%)		
At 24 months, has heard of Active Ottumwa	72 (99%)		
At 24 months, has participated in an event with a PAL	10 (14%)		
At 24 months, reported increasing PA (as an individual or in family) as a result of seeing Active Ottumwa messages	42 (59%)		
At 24 months, has heard of any specific Active Ottumwa events (e.g. walking groups, Zumba, tai chi)	72 (99%)		
Reported participating in any specific Active Ottumwa events (e.g. walking groups, Zumba, tai chi)	14 (19%)		

^a^Compositional PA values

We performed Pillai-Bartlett tests testing all variables for associations with change in PA (Supplemental file 4, table A). There was a significant global change in PA (*P* = *0.03*) corresponding to a decrease in SA and an increase in LPA. Age, gender, and education were not significantly associated with change in PA between baseline and 24 months. Initial levels of PA were significantly associated with change in PA composition (initial MVPA *P* = 0.04; initial SA *P* = 0.01). Specifically, a higher level of MVPA at baseline was associated with a higher follow-up level of LPA, and a higher level of SA at baseline was associated with a lower follow-up level of SA and higher follow-up level of LPA.

Based on the results of this analysis, we estimated the expected change after two years’ of exposure to the Active Ottumwa intervention in terms of PA composition and minutes of PA out of a 16 h day (Table 3). An average resident of the community would be expected to reduce SA by 45 min, increase LPA by 35 min, and increase MVPA by 9.3 over 24 months.

**Table 3:** Expected change over 24 months per person with median baseline SA and PA

	***Sedentary***	***Light***	***Moderate/Vigorous***
**Median**			
Baseline^a^	0.73	0.15	0.13
24 months follow up^a^	0.69	0.19	0.12
∆Minutes of 16 h day	-37	44	-6.5
**Mean**			
Baseline^a^	0.75	0.17	0.08
24 months follow up^a^	0.70	0.21	0.09
∆Minutes of 16 h day	-45	35	9.3

^a^Compositional score

### Cross-sectional community survey

Both the 2013 and 2018 surveys had an overrepresentation of females and adults over the age of 45 years that differed from Ottumwa’s population (*P* < *0.001* for both) (See Supplemental file 5 Table B). Latinos were slightly underrepresented in the 2013 survey (*P* = *0.01*) but not in the 2018 survey (*P* = *0.68*).

We saw an estimated increase of 1.45% in the mean GPAQ MVPA composition, or roughly 14 min in a 16-h day, with a 95% confidence interval of -1.94% – 4.84%, or -19 – 46 min, change. This increase, however, was not statistically significant (*P* = 0.20) (See Supplemental file 6, Table C). Meanwhile, the population mean of the recalibrated GPAQ MVPA composition was estimated to have increased by 0.72% between 2013 and 2018, or roughly 7 min in a 16-h day, with a 95% confidence interval of a 0.1%—1.34%, or 1 – 13 min in a 16-h day, increase. While this increase was smaller than that of the raw GPAQ MVPA, it was statistically significant (*P* = 0.01).

The distribution of raw GPAQ values differed considerably from recalibrated GPAQ values. We compared these distributions graphically (see Supplemental file 3 Figure A) with the longitudinal cohort, where we obtained both GPAQ and accelerometry measurements [22]. The strong similarity between the GPAQ values of the cohort and 2013/2018 community surveys as well as the similarity between the recalibrated GPAQ values of the surveys with the accelerometry measurements of the cohort provides visual evidence in support of an improvement in accuracy from using the recalibrated GPAQ rather than raw GPAQ values [26].

## Discussion

Active Ottumwa was a population-based PA intervention that implemented evidence-based strategies to promote PA in a rural micropolitan community. We evaluated the effectiveness of the intervention at the individual-behavioral and population levels, using cohort and cross-sectional methods. Both sources of data showed an increase in PA during the study period. In a cohort of participants whose PA was directly measured at multiple time points over a 24-month period, we found that exposure to the intervention, measured by the time participants in the cohort study were exposed to the Active Ottumwa implementation, was associated with decreases in SA and increases in LPA and MVPA. In two cross-sectional surveys, conducted before and after the intervention was implemented, we found significant increases in minutes of self-reported MVPA. These findings are consistent with the hypothesis that this evidence-based community intervention was effective at supporting PA changes at individual- and population-levels.

The findings from this study add to small but growing evidence that interventions implemented in and tailored to rural settings to promote PA are effective at increasing PA. The replication and adaptation of evidence-based strategies to promote PA which were developed and tested for urban settings also suggests the internal and theoretical consistency of these strategies (i.e., lay health advisors, health communications). Our study adds to the external validity of these strategies [30]. The findings of the study demonstrate that it is necessary, and effective to adapt and implement evidence-based PA interventions mostly developed in urban settings to rural communities, in ways that are consistent with local contexts and values. Essential factors that strengthen the study design and implementation included our (1) long-term engagement with diverse stakeholders across the community; (2) population-based approach and community partner involvement in the adaptation and implementation of the intervention; (3) use of evidence-based strategies to promote PA at the individual, social and built environment levels; and (4) the use of lay health advisors. We consider these factors to be necessary strategies to successfully implement interventions within rural communities. In our case, we followed a CBPR approach to engage with community members from the inception of the research idea, the implementation, evaluation and dissemination of findings was critical and successful in rural context.

The study and its findings have practical implications for public health and population health. Careful consideration of adaptation of strategies developed in urban areas to the context of a rural community can help to facilitate implementation and dissemination of those strategies. Although results were modest in the increase in PA, there were significant improvements at the population level, and even low levels of PA has been shown to be beneficial for health and reducing mortality risks [7]. The magnitude of the change in PA is similar to other studies in rural and urban areas. In our case, we measured significant increases in LPA and decreases of sedentary behavior, which indicates that sedentary individuals were motivated and supported by the intervention’s activities to become somewhat active, and therefore improving their health almost immediately. Our findings have internal validity and are externally valid as well. We collected both objective and subjective PA data prospectively from a randomly selected cohort of residents and through cross-sectional population-based surveys. These two separate but complementary measures yield similar and complementary results, confirming the validity and implications of the results. Community-engaged interventions to successfully increase PA opportunities and reduce sedentary behavior in rural micropolitan communities have the potential to reduce health disparities in PA and related health outcomes that many communities experience. Support and funding for community-engaged research can build capacity among communities to implement evidence-based interventions for chronic disease.

The study has some limitations. First, direct measurement of PA through an accelerometer may induce a form of bias known as the Hawthorne effect, where participants engage in more PA than normal when wearing an accelerometer. This may upwardly bias each participant’s observed PA. However, because the outcome of the study is change in PA over time, this bias should not affect our study findings if the amount of bias is consistent throughout the study period. It is also possible that this effect decays over time, in which case our results would be biased towards the null hypothesis of no intervention effect. Second, selection bias, including loss to follow may also have been introduced. To account for differences in PA trajectories over the study period between individuals who ceased participation after the baseline and individuals who remained in the study, we controlled for age, gender and education. A third limitation was the repeated measures design and a “regression to the mean” effect. If unaccounted for, these effects might drown out the overall signal in the data or possibly lead to incorrect interpretations of the data analysis. To address this, we adjusted for each subject’s baseline PA when analyzing change in their PA. Fourth, there was no formal analysis done to disaggregate the data based on sex/gender, race/ethnicity. Fifth, there was a sizeable amount of loss to follow-up, which makes the results less likely to be generalized.

Our study had several strengths. First, the quasi-experimental study design that included cohort and cross-sectional measurements of individuals in the community where the intervention was implemented, was a robust test of external validity of the study. These methods allowed us to evaluate the impact of the intervention at multiple levels of behavior influence. Second, using different methods of data collection, both objective (cohort) and self-reported (community) measures of PA, allowed us to compare and contrast changes observed at the individual and population level. Interpreting the findings based on 16-h awake time allows for the inclusion of all types and experiences of PA for participants in this rural area. Our calibration study conducted in the first year [26] added strength to the reliability and validity of both our PA measures. This process provides the community the opportunity to contextualize the evidence to the resources and needs of this micropolitan rural community. Finally, the study design allows for intervention activities to continue in the community and this approach enhances sustainability to further increase PA, particularly in rural areas.

## Conclusions

More than ever rural communities need support and attention to address the historical and structural inequities contributing to their persistent health disparities. The Active Ottumwa partnership used a CBPR approach to adapt and implement evidence-based strategies to promote PA across the community, building off of strengths in the community. The partnership leveraged the social environment in the form of the social networks of LHAs, and the built environment in the form of community infrastructure and community organizations to implement the intervention. The evidence generated provides support to replicate this type of intervention in other communities. It is particularly important for rural communities where less evidence is available and available evidence of potential community-based interventions can be scaled through implementation science. The Active Ottumwa intervention can serve as a model to promote healthier behaviors across communities.

### Supplementary Information

**Supplementary Material 1.**

## Data Availability

The datasets generated and/or analysed during the current study are not publicly available because permission is needed from the Community Advisory Board but are available from the corresponding author on reasonable request.

## Abbreviations

PA: Physical Activity
LHA: Lay Health Advisors
SA: Sedentary Activity
LPA: Light Physical Activity
MVPA: Moderate-to-Vigorous Physical Activity
clr: Centered log ratio
MANOVA: Multivariate Analysis Of Variance
GPAQ: Global Physical Activity Questionnaire (GPAQ)
ACS: American Community Survey
CBPR: Community-Based Participatory Research

## References

[1] Centers for Disease Control Prevention, National Center for Chronic Disease Prevention, Health Promotion Division of Nutrition, Physical Activity, and Obesity. Data, trends and maps. https://www.cdc.gov/nccdphp/dnpao/data-trends-maps/index.html. Accessed 2 Dec 2019.

[2] Center for Disease Control, National Center for Health Statistics. FastStats – exercise or physical activity. Updated 2019–09–04. https://www.cdc.gov/nchs/fastats/exercise.htm. Accessed 13 Dec 2019.

[3] Blackwell D, Clarke T. State variation in meeting the 2008 federal guidelines for both aerobic and muscle-strengthening activities through leisure-time physical activity among adults aged 18–64: United States, 2010–2015. National Health Statistics Report. 2018. https://www.cdc.gov/nchs/data/nhsr/nhsr112.pdf. 30248007

[4] Sharpe PA, Jackson KL, White C (1997). Effects of a one-year physical activity intervention for older adults at congregate nutrition sites. Gerontologist.

[5] Thorp A, Kingwell B, Sethi P, Hammond L, Owen N, Dunstan D (2014). Alternating bouts of sitting and standing attenuate postprandial glucose responses. Med Sci Sports Exerc.

[6] Buman MP, Hekler EB, Haskell WL (2010). Objective light-intensity physical activity associations with rated health in older adults. Am J Epidemiol.

[7] Zhao M, Veeranki SP, Li S, Steffen LM, Xi B (2019). Beneficial associations of low and large doses of leisure time physical activity with all-cause, cardiovascular disease and cancer mortality: a national cohort study of 88,140 US adults. Br J Sports Med.

[8] Department of Health, Physical Activity, Health Improvement, and Protection (2011). Start active, stay active.

[9] Hamer M, Stamatakis E, Steptoe A (2014). Effects of substituting sedentary time with physical activity on metabolic risk. Med Sci Sports Exerc.

[10] Gando Y, Yamamoto K, Murakami H (2010). Longer time spent in light physical activity is associated with reduced arterial stiffness in older adults. Hypertension.

[11] Dunstan DW, Kingwell BA, Larsen R (2012). Breaking up prolonged sitting reduces postprandial glucose and insulin responses. Diabetes Care.

[12] Healy GN, Dunstan DW, Salmon J (2007). Objectively measured light-intensity physical activity is independently associated with 2-h plasma glucose. Diabetes Care.

[13] Loprinzi PD, Lee H, Cardinal BJ (2015). Evidence to support including lifestyle light-intensity recommendations in physical activity guidelines for older adults. Am J Health Promot.

[14] Martin SL, Kirkner GJ, Mayo K, Matthews CE, Durstine JL, Hebert JR (2005). Urban, rural, and regional variations in physical activity. J Rural Health.

[15] Community Preventive Services Task Force. Increasing physical activity. https://www.thecommunityguide.org/topic/physical-activity.

[16] U.S. Department of Health and Human Services (2018). Physical activity guidelines for Americans.

[17] Meyer MRU, Perry CK, Sumrall JC, et al. Physical activity–related policy and environmental strategies to prevent obesity in rural communities: a systematic review of the literature, 2002–2013. Prev Chronic Dis. 2016;13(E03). 10.5888/pcd13.150406. 10.5888/pcd13.150406 PMC4707945 26741997

[18] Curran GM, Bauer M, Mittman B, Pyne JM, Stetler C (2012). Effectiveness-implementation hybrid designs: combining elements of clinical effectiveness and implementation research to enhance public health impact. Med Care.

[19] U.S. Census Bureau (2019). QuickFacts.

[20] County Health Rankings. Wapello County, Iowa. University of Wisconsin Population Health Institute, Robert Wood Johnson Foundation. https://www.countyhealthrankings.org/app. Accessed 12–4–2019.

[21] Israel BA, Schulz AJ, Parker EA, Becker AB, Community-Campus Partnerships for Health (2001). Community-based participatory research: policy recommendations for promoting a partnership approach in health research. Educ Health (Abingdon).

[22] Baquero B, Kava CM, Ashida S, et al. Active Ottumwa: adapting evidence-based recommendations to promote physical activity in a micropolitan new destination community. Int J Environ Res Public Health. 2018;15(5). 10.3390/ijerph15050917. 10.3390/ijerph15050917 PMC5981956 29734709

[23] American Association for Public Opinion Research Cell Phone Task Force (2010). New considerations for survey researchers when planning and conducting RDD telephone surveys in the U.S. with respondents reached via cell phone numbers.

[24] Harris PA, Taylor R, Minor BL (2019). The REDCap consortium: building an international community of software platform partners. J Biomed Inform.

[25] ActiGraph. ActiGraph link. https://www.actigraphcorp.com/actigraph-link/. Accessed 12–4–2019.

[26] Metcalf KM, Baquero BI, Garcia MLC (2018). Calibration of the global physical activity questionnaire to Accelerometry measured physical activity and sedentary behavior. BMC Public Health.

[27] Aitchison J. The statistical analysis of compositional data. Springer Netherlands: Blackburn Press; 2003.

[28] Lohr SL (2010). Sampling: design and analysis.

[29] World Health Organisation. Global Physical Activity Questionnaire (GPAQ) https://www.who.int/ncds/surveillance/steps/resources/GPAQ_Analysis_Guide.pdf.

[30] Walsh SM, Meyer MRU, Gamble A, Patterson MS, Moore JB (2017). A systematic review of rural, theory-based physical activity interventions. Am J Health Behav.

