# Supplementary material for: Effectiveness of implementing evidence-based approaches to promote physical activity in a Midwestern micropolitan area using a quasi-experimental hybrid type I study design
Source: BMC Public Health. 2024 Apr 19;24:1082. doi: 10.1186/s12889-024-18523-9 (PMC11027347; doi:10.1186/s12889-024-18523-9)
Supplement: Supplementary file 1 — Supplementary Material 1. [file 12889_2024_18523_MOESM1_ESM.docx]

**Supplementary file 1: Cohort Study – Analytic Approach – Additional Information**

*Step 1.* We evaluated change in PA composition normalized by the amount of time each individual was exposed to the Active Ottumwa intervention. Because typical arithmetic operations are not appropriate on compositional variables lying on a simplex,^[[1]](#footnote-1)^ we instead computed the compositional slopes using the group operations on the simplex that are the direct analogs of subtraction and division that we are more accustomed to applying. Using notation commonly applied to compositional analysis, the compositional slope for the $i^{th}$ individual can be computed in the following way:

$$Slope_{i}=\frac{1}{T_{i}}\otimes\left( \boldsymbol{y}_{i1}-\boldsymbol{y}_{i0} \right),$$

where $T_{i}$ is the amount of time $i$ was exposed to the intervention, and $\boldsymbol{y}_{i1}$ ($\boldsymbol{y}_{i0}$) is the compositional PA variable for individual $i$ at their 24-month follow-up (baseline) visit, i.e.,

$$\boldsymbol{y}_{i1}=\left( \frac{\# minutes SA}{Total \# minutes},\frac{\# minutes LPA}{Total \# minutes},\frac{\# minutes MVPA}{Total \# minutes} \right).$$

If there is no change from baseline to 24 month follow-up, the compositional slope will equal (1/3, 1/3, 1/3). Arithmetically, the compositional slope can be computed in the following way:

$$Slope_{i}\mathcal{=C}\left( \left( \frac{\boldsymbol{y}_{i1,SA}}{\boldsymbol{y}_{i0,SA}} \right)^{\frac{1}{T_{i}}},\left( \frac{\boldsymbol{y}_{i1,LPA}}{\boldsymbol{y}_{i0,LPA}} \right)^{\frac{1}{T_{i}}},\left( \frac{\boldsymbol{y}_{i1,MVPA}}{\boldsymbol{y}_{i0,MVPA}} \right)^{\frac{1}{T_{i}}} \right),$$

where $\mathcal{C}$ indicates the “closure” of a vector, which simply means to normalize the vector such that the sum of its elements will equal 1.

*Step 2.* To allow for the implementation of standard statistical procedures, we performed the centered log ratio (clr) transformation. This transformation is defined to be

$$\left( clr\left( SA \right),clr\left( LPA \right),clr\left( MVPA \right) \right)=\left( \log\left( \frac{SA}{g} \right),\log\left( \frac{LPA}{g} \right),\log\left( \frac{MVPA}{g} \right) \right),$$

where $g$ is the geometric mean of (*SA*, *LPA*, *MVPA*).

*Step 3.* An informal representation of the MANCOVA model can be given as

$$\left( clr\left( \Delta SA \right),clr\left( \Delta LPA \right),clr\left( \Delta MVPA \right) \right)=Overall change+Age+Gender+Education+clr\left( SA_{baseline} \right)+clr\left( MVPA_{baseline} \right).$$

Note that due to the constraint that the clr of the baseline PA will sum to zero, also including baseline LPA as a covariate would lead to a non-identifiable model.

*Step 4.* Given baseline measurements $BL=(SA_{baseline},LPA_{baseline},MVPA_{baseline})$ and compositional slope $S=(\Delta SA,\Delta LPA, \Delta MVPA)$, we can compute the final measurement over a period of length $T$ by

$$\mathcal{C}\left( \left( T\otimes S \right)\oplus BL \right)$$

or arithmetically

$$\mathcal{C}\left( \left( \Delta SA \right)^{T}SA_{baseline} , \left( \Delta LPA \right)^{T}LPA_{baseline},\left( \Delta MVPA \right)^{T}MVPA_{baseline} \right).$$

**Supplemental file 2: Community Survey** **– Analytic Approach – Additional Information**

We estimated the true population clr-transformed compositional PA variables consisting of SA and MVPA using weighted averages, where the weights were determined using age, gender, and ethnicity. We used a Wald test on the null hypothesis that there was no difference in PA levels between 2013 and 2018.

For confidence intervals, we used the delta method. Let $\omega_{t}$ ($\hat{\omega}_{t}$) denote the true (estimated) population clr value at time $t$, $t\in\{2013,2018\}$, i.e.,

$$\omega_{t}:=\log\left( \frac{\theta_{t,MVPA}}{\sqrt{\theta_{t,SA}\theta_{t,MVPA}}} \right), \hat{\omega}_{t}:=\log\left( \frac{\hat{\theta}_{t,MVPA}}{\sqrt{\hat{\theta}_{t,SA}\hat{\theta}_{t,MVPA}}} \right),$$

where $\theta_{t,MVPA}$ ($\theta_{t,SA}$) is the median proportion of time the population spends in MPVA (SA). Then using the delta method, we have that

$$\hat{\theta}_{2018,MVPA}-\hat{\theta}_{2013,MVPA} \overset{\cdot}{\sim} N\left( \theta_{2018,MVPA}-\theta_{2013,MVPA},V \right),$$

where

$$V:= 4\left( \hat{\theta}_{2018,MVPA}^{2}\left( 1-\hat{\theta}_{2018,MVPA} \right)^{2}\hat{\sigma}_{2018}^{2}+\hat{\theta}_{2013,MVPA}^{2}\left( 1-\hat{\theta}_{2013,MVPA} \right)^{2}\hat{\sigma}_{2013}^{2} \right),$$

and $\hat{\sigma}_{t}^{2}$ is the estimated variance of $\hat{\omega}_{t}$. This normal distribution can then be used to create approximate confidence intervals for the change in PA on the compositional scale.

| **Supplemental file 3:**  **Figure A**. Distributions of the proportion of PA spent in MVPA according to the raw GPAQ values (gray) and rGPAQ calibrated values (black) for the community surveys. For the longitudinal cohort, the black curve instead represents the MVPA according to the accelerometry data. | | |
| --- | --- | --- |
| 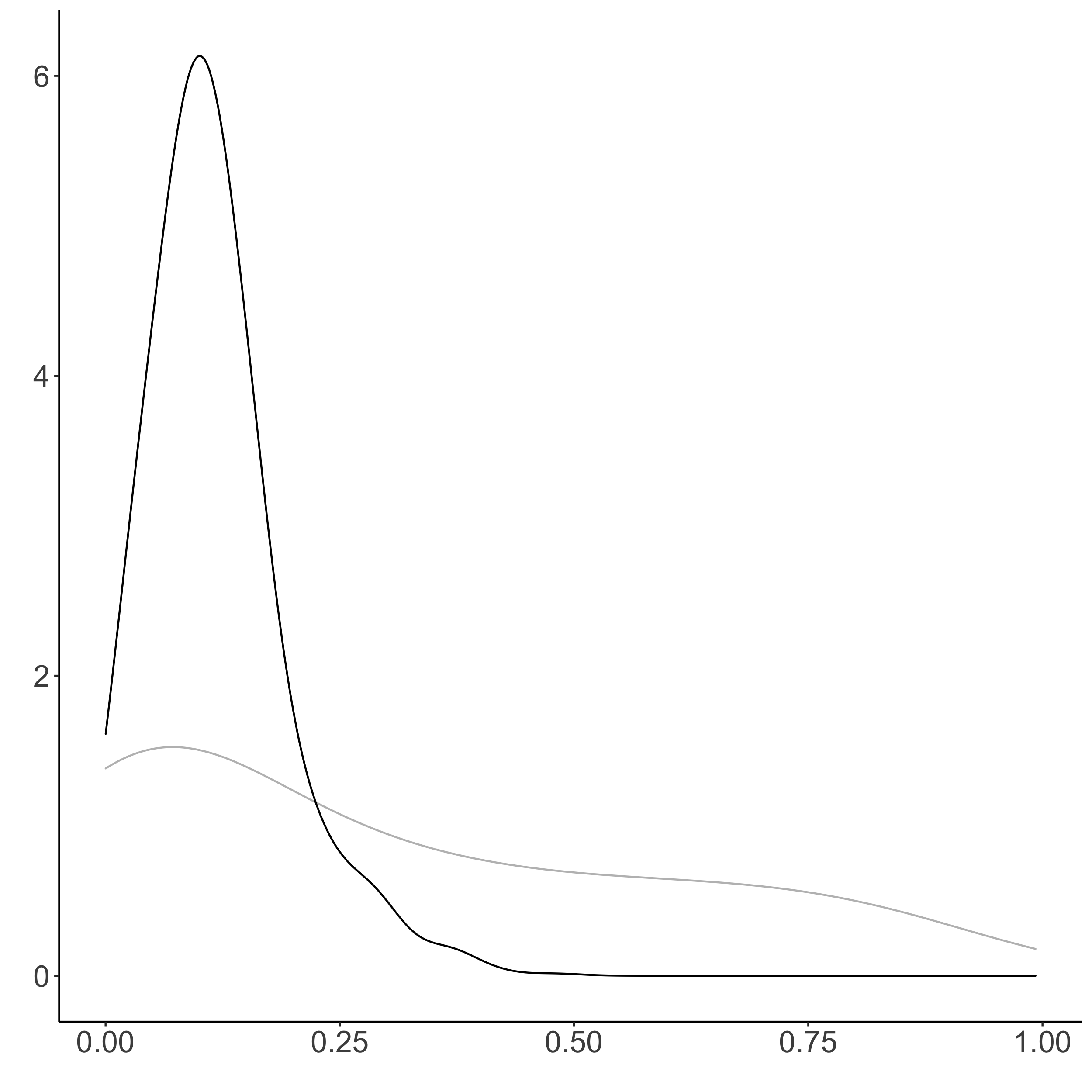 | 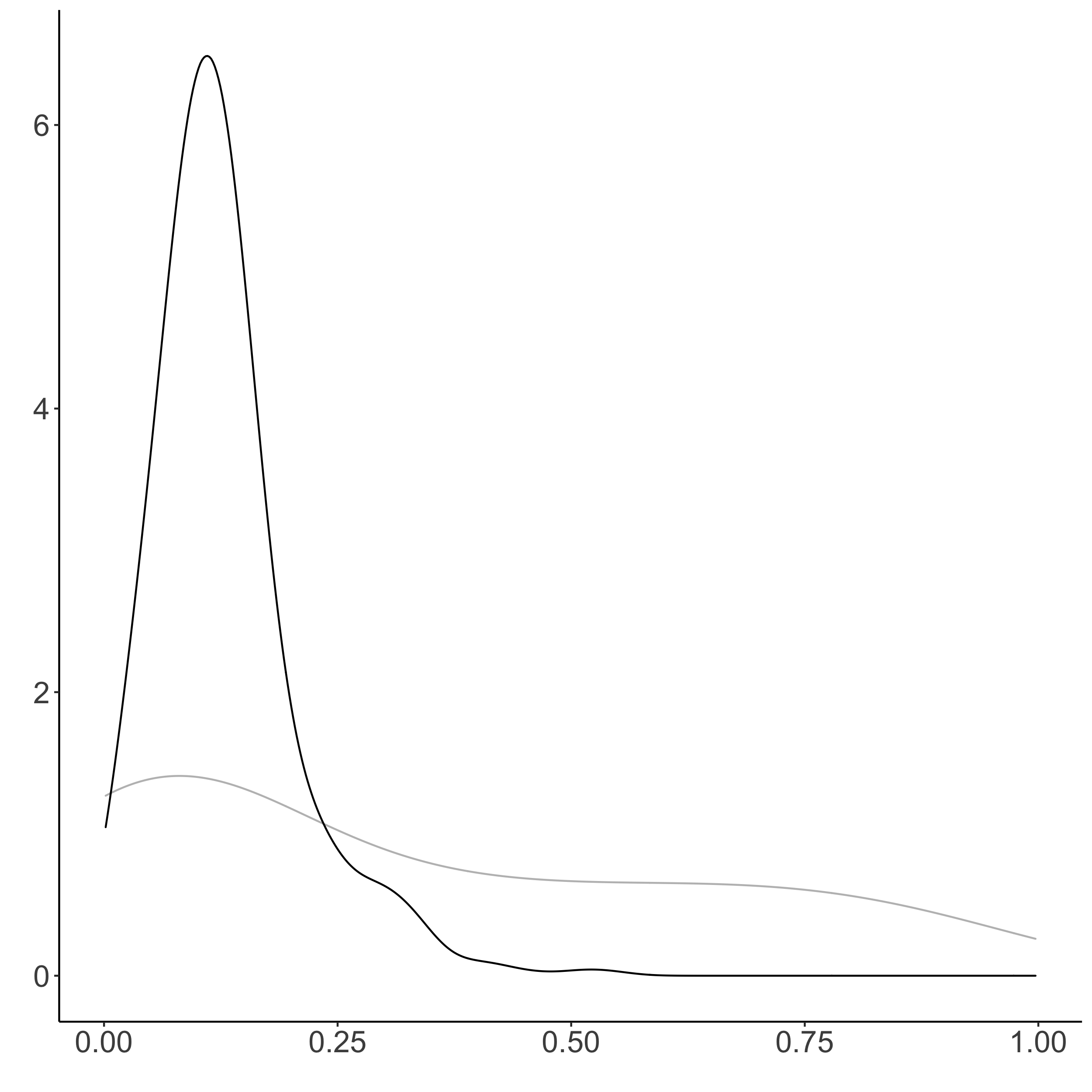 | 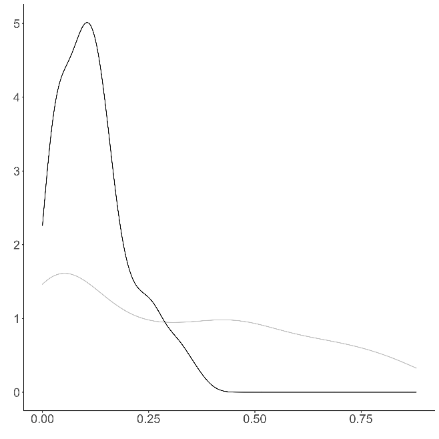 |
| (a) 2013 Community Survey | (b) 2018 Community Survey | (c) Longitudinal Cohort (baseline) |

**Supplemental file 4:**

**Table A.** MANCOVA model run on the clr PA slope variables using the longitudinal cohort data. Confidence intervals are at the 90% level.

|  | ***p-value*** | ***Sedentary*** | ***Light*** | ***Moderate/Vigorous*** |
| --- | --- | --- | --- | --- |
| Overall change | 0.03 | -0.07  (-0.14, -0.01) | 0.06  (0.02, 0.10) | 0.02  (-0.05, 0.08) |
| Age | 0.47 | 0.03  (-0.02, 0.076) | 0.00  (-0.03, 0.03) | -0.03  (-0.08, 0.02) |
| Gender (Male) | 0.27 | -0.04  (-0.08, 0.00) | 0.00  (-0.02, 0.03) | 0.03  (-0.01, 0.08) |
| Education level | 0.20 |  |  |  |
| 8^th^ grade or less |  | -0.15  (-0.28, -0.01) | 0.01  (-0.07, 0.09) | 0.14  (0.01, 0.27) |
| Some high school |  | -0.08  (-0.21, 0.05) | 0.05  (-0.03, 0.13) | 0.03  (-0.10, 0.16) |
| Graduated High School |  | 0.17  (0.08, 0.26) | -0.04  (-0.09, 0.02) | -0.13  (-0.22, -0.05) |
| Initial Moderate or Vigorous | 0.04 | -0.05  (-0.17, 0.06) | 0.14  (0.07, 0.21) | -0.08  (-0.20, 0.03) |
| Initial Sedentary | 0.01 | -0.12  (-0.23, -0.01) | 0.13  (0.07, 0.20) | -0.01  (-0.12, 0.10) |

**Supplemental file 5:**

**Table B**. Demographics of community survey participants. n (%): Ottumwa, Wapello,, Iowa, 2013-2019

|  | **Community Survey 2013** | **Ottumwa** **Population 2013** | **Community Survey 2018** | **Ottumwa** **Population 2017** |
| --- | --- | --- | --- | --- |
|  | N (%) | N (%) | N (%) | N (%) |
| Gender |  |  |  |  |
| Female | 650 (62%) | 10,132 (53%) | 525 (59%) | 9,610 (51%) |
| Male | 392 (38%) | 9,087 (47%) | 370 (41%) | 9,059 (49%) |
| Age |  |  |  |  |
| Age 18-29 | 110 (11%) | 4,404 (23%) | 114(13%) | 4,349 (23%) |
| Age 30-44 | 159 (15%) | 4,693 (24%) | 189 (21%) | 4,610 (25%) |
| Age 45+ | 773 (74%) | 10,122 (53) | 592 (66%) | 9,710 (52%) |
| Latino | 70 (7%) | 1,749 (9%) | 100 (11%) | 2,180 (12%) |

Data sources: Anonymous.

**Supplemental file 6:**

**Table C.** Change in the mean of GPAQ MVPA composition between 2013-2018: Ottumwa*, Wapello , Iowa, 2013-2019.*

|  | Raw GPAQ MVPA  Compositional Means | recalibrated GPAQ MVPA Compositional Mean |
| --- | --- | --- |
| 2013 | 0.1863 | 0.1162 |
| 2014 | 0.2008 | 0.1234 |
| Change (2018-2013)  95% CI  p-value | 0.0145 (-0.0194-0.0484)  0.20 | 0.0072 (0.0010-0.0134)  0.01 |

1. The K-simplex is the space of K+1 positive values under the constraint that these positive values sum to one. An individual’s physical activity compositional variable thus lies on the 2-simplex, as the proportion of time an individual spends in SA, LPA, and MVPA must sum to one. [↑](#footnote-ref-1)
